# A Detroit Student-Run Free Clinic’s Management of Select Chronic Diseases

**DOI:** 10.7759/cureus.26701

**Published:** 2022-07-09

**Authors:** Serina B Beydoun, Anna H Lee, Leigh Durudogan, Virginia Kaufman, Morgan Potter, Firas Askar, Charles Tsouvalas, Brian Reed, Robert L Sherwin

**Affiliations:** 1 Pediatrics, Children's Hospital of Michigan, Detroit, USA; 2 Internal Medicine, University of California-Los Angeles Medical Center, Los Angeles, USA; 3 Obstetrics and Gynecology, Beaumont Hospital, Royal Oak, USA; 4 Department of Obstetrics and Gynecology, Detroit Medical Center, Detroit, USA; 5 Emergency Medicine, Icahn School of Medicine at Mount Sinai, New York, USA; 6 Internal Medicine, Henry Ford Health System, Detroit, USA; 7 Internal Medicine, Kaiser Permanente, Los Angeles, USA; 8 Department of Emergency Medicine, Wayne State University School of Medicine, Detroit, USA; 9 Department of Emergency Medicine, Detroit Medical Center, Detroit, USA

**Keywords:** obesity, underserved, diabetes mellitus, hypertension, student-run free clinic

## Abstract

Aim: The Cass Clinic is a student-run free clinic in Detroit, Michigan that treats chronic diseases including hypertension (HTN), diabetes mellitus (DM), and obesity. Our study aims to quantify the effectiveness of our clinic in managing chronic diseases.

Subject and methods: This study assessed selected health outcomes for 137 patients who visited our clinic between September 1, 2017 and August 31, 2018 based on initial and most recent surrogate markers including manual blood pressure, hemoglobin A1c (HbA1c), and body mass index (BMI) recorded in the clinic’s medical record system dating back to 2012.

Results: Patients were divided into two groups: occasionally seen patients (OSP) and frequently seen patients (FSP). FSP with HTN had systolic blood pressure (SBP) decreased by an average of 14.1 mmHg and diastolic blood pressure (DBP) decreased by 9.8 mmHg, which were statistically associated with the number of clinic visits. Additionally, all patients treated at Cass Clinic saw a decrease in their HbA1c and BMI. HbA1c in OSP decreased by 0.50%. HbA1c in the FSP decreased by 1.7%. Patients with at least two recorded BMIs (n=73) saw a decrease of 0.13 kg/m^2^.

Conclusion: The data from our analysis support that a student-run free clinic model like Cass Clinic provides long-term value for patients who frequently utilize the clinic. These clinics also act as an important resource for the community by making positive strides toward better health in multiple measurable outcomes, including HTN and DM management.

## Introduction

This article was previously presented as a poster at the American Medical Association Research Symposium on November 15, 2019. Hypertension (HTN), type II diabetes mellitus (DM), and obesity are highly prevalent in the U.S. and greatly contribute to morbidity and mortality. Data collected by the Center for Disease Control and Prevention (CDC) revealed that approximately 75 million U.S. adults between 2011 and 2012 had HTN, which equates to one in every three adults [1]. In 2015, more than 100 million U.S. adults were living with DM or prediabetes [2]. In 2015-2016, obesity, which affected 93.3 million U.S. adults, was prevalent in 39.8% of the population [3]. Individuals with these chronic diseases are at an increased risk for heart disease and stroke, which are the leading causes of death in the U.S. The estimated combined medical costs of these conditions result in hundreds of billions of dollars each year. These diseases place a significant strain on our economy and the overall health of our society.

Treating these chronic conditions is important in reducing this burden. However, whether a patient can achieve and maintain disease control is dependent on several socioeconomic factors, including their access to affordable healthcare. The U.S. had 13.7% uninsured and 29% underinsured adults in the fourth quarter of 2018, both at their highest level since the first quarter of 2014 [4,5]. Many uninsured and underinsured patients rely on safety-net hospitals, non-profit or faith-based organizations, and free clinics for their medical needs. In 2018, free health care was provided to over 34,044 uninsured patients in Michigan via various free clinics, including student-run free clinics [6]. By managing HTN, DM, and obesity in student-run free clinics, such as the Cass Clinic, there is potential to dramatically improve the health of uninsured and underinsured patients.

Cass Clinic is a student-run free clinic organization operating two sites in Detroit. It has served underinsured and uninsured patients since the late 1970s. The clinic primarily treats chronic diseases, including HTN, DM, and obesity. During a typical patient visit, medical students conduct an interview, perform a focused physical exam, and, when appropriate, obtain certain laboratory tests, including glycosylated hemoglobin A1c (HbA1c), lipid panel, and blood glucose readings. The student team then presents the case to an attending physician and, together, they come up with a treatment plan, which includes medications, lifestyle modifications, patient education, and referrals.

All Cass Clinic patients have their vital signs, height, and weight checked at every visit. BMI calculation allows for proper nutrition and exercise counseling. Cass Clinic patients with HTN have their blood pressure manually checked by either a medical student or a volunteer nurse practitioner. Medications are then adjusted accordingly, and they are subsequently given a one-month refill on their antihypertensive medications. During the visit, they will also receive counseling on improving their nutrition, smoking cessation, and the importance of exercise. Cass Clinic patients with DM are provided glucometers and asked to measure their blood glucose at least twice a day, which is then reviewed at each visit. A fasting or random glucose test is checked, and patients receive a one-month refill on their insulin, needles, syringes, and glucometer test strips, in addition to access to a diabetes nurse educator. Cass Clinic also hosts several other student organizations, including FreshRx, which provides patients with a prescription to eat more fruits and vegetables, which can be filled at partnering farm stands or markets, and Sight Savers, which provides free eye exams, glaucoma screenings, and prescription glasses. In addition to chronic disease management, patients receive care for acute illnesses, allergies, and asthma. In 2017, Cass Clinic facilitated over 650 patient encounters provided by over 1,688 volunteer hours from undergraduate, medical, and other healthcare-associated graduate students.

Nearly, 38% of Detroit’s approximate 670,000 residents lived below the poverty line in 2017, with 13.8% of the population under 65 being uninsured [7]. It is well documented that health outcomes and chronic disease management are negatively affected by poverty and lack of insurance coverage, making our clinic an important resource for patients who would otherwise go untreated. While providing free medical care for this population seems valuable, the impact of Cass Clinic on chronic disease management has never been formally quantified. There have been several studies showcasing that HTN management in student-run free clinics is on par with national data [8,9]. However, limited research exists concerning the treatment and outcomes of DM in these clinics, and studies regarding counseling and improvements among the obese populations are rare. Our methodology is unique in that we were able to individually assess the HTN, DM, and obesity management of Cass Clinic patients.

The study aims to confirm whether Cass Clinic improved selected health outcomes for its patients with chronic diseases including HTN, DM, and overweight/obesity based on surrogate markers including manual blood pressure, HbA1c, and body mass index (BMI), respectively, that have been recorded in the clinic’s electronic medical record (EMR) system. We also looked for differences in outcomes based on visit frequency; that is, whether a patient was a frequently seen patient (FSP) or an occasionally seen patient (OSP) in the clinic. This study will help Cass Clinic and other student-run free clinics better understand the Detroit population that utilizes our clinics and how to best provide care to our patients.

## Materials and methods

Cass Clinic has student teams document all patient encounters in an EMR system that has been in use since 2012, PracticeFusion©, and patient charts are subsequently reviewed by the clinic EMR coordinator and signed as a quality control measure. This study was conducted by the Cass Clinic coordinators. There were no exclusion criteria for this study. Patients were included if they had been treated in our clinic between September 1, 2017 and August 31, 2018. This time period was chosen as it was before the COVID-19 pandemic caused a temporary shutdown of the clinic. After selecting patients, patient data including patient date of birth, age, gender at birth, ethnicity/race, current or history of smoking, diagnosis of DM or HTN, first encounter date, most recent encounter date, medication for DM or HTN at any time as a Cass Clinic patient, the total number of days between first and most recent encounters, the total number of visits documented in the EMR, first and most recent documented blood pressure, first and most recent documented HbA1c, and first and most recent documented BMI were collected via chart review (Table 1). These variables were all chosen as they are consistently documented in each patient's chart and can act as valid surrogate markers for the management of chronic diseases. The Cass Clinic EMR does not have a designated area for HbA1c documentation. Instead, the result of an HbA1c lab test is written in the encounter note. The patient’s first encounter date may have occurred as early as 2012, when the Cass Clinic transitioned to the current EMR system from paper-based documentation. Data from 2012 to the end of the study period on August 31, 2018 was included. Patients who were only seen once were excluded.

**Table 1:** Variables and data collected DM: diabetes mellitus, HTN: hypertension, HbA1c: hemoglobin A1c, BMI: body mass index

Variable	Data collected
Date of birth	Month/day/year (age)
Gender at birth	Male or female
Ethnicity/race	African American, White, Hispanic/Latino, Native American, Asian American, other
Smoker at anytime	Yes or no
Type 2 DM diagnosis	Yes or no
HTN diagnosis	Yes or no
First encounter date	Month/day/year
Most recent encounter date	Month/day/year
At any time DM medication	Yes or No
At any time HTN medication	Yes or No
Total number of days between first encounter and last encounter	Total number of days
Total number of visits	Total number of visits
First documented blood pressure	Systolic BP/diastolic BP
First documented HbA1c	HbA1c number
First documented BMI	BMI number
Most recent documented blood pressure	Systolic BP/diastolic BP
Most recent documented HbA1c	HbA1c number
Most recent documented BMI	BMI number

Patients were considered to have HTN if they reported a prior physician diagnosis of HTN, previous use of HTN medication, or if they had two separate readings of either systolic blood pressure (SBP) >140 mmHg or diastolic blood pressure (DBP) >90 mmHg, which is consistent with the Seventh Report of the Joint National Committee on Prevention, Detection, Evaluation, and Treatment of High Blood Pressure guidelines in 2012, when our EMR was first introduced and many of our frequently seen patients’ first blood pressure readings were recorded. Similarly, patients were considered to have DM if they reported a prior physician diagnosis of DM, previous use of DM medication, or HbA1c >6.5%. A unique patient identification number identified each patient, and all information was input into an Excel spreadsheet (Microsoft Excel, Microsoft® Corp., Redmond, WA). The difference (referred to as Delta in the results section) in the patient’s initial and most recent measurements for systolic blood pressure and diastolic blood pressure, BMI, and HbA1c were then calculated to determine the change in each surrogate marker as a reflection of chronic disease management.

Initial screening found 252 patients who were seen during our study period. Patients who were only seen once were excluded from the analysis (n=115). Charts were reviewed for the remaining 137 patients. The patients were then analyzed based on overall clinic use rate, diagnosis of HTN, and diagnosis of DM. Based on the overall clinic use rate, they were divided into "OSP" and "FSP" based on how many total visits to the clinic they made. The median number of visits for all patients included in the analysis was nine visits. This served as our cut-off, establishing OSP as those with a total of two to nine visits and FSP as those who have more than nine visits. Patients were also separated by a diagnosis of either HTN or DM and again stratified by utilization of the clinic. There were 110 patients diagnosed with HTN. The median visit count was 15 for patients diagnosed with HTN and this served as our cut-off with OSP having a total of two to 15 visits and FSP having more than 15 visits. There were 43 patients diagnosed with DM. The median visit count was 13 for patients diagnosed with DM, and this served as our cut-off, with OSP having a total of two to 13 visits and FSP having more than 13 visits.

Analysis was performed with statistical analysis systems (SAS) and included the use of a t-test, Wilcoxon Rank Sum t-test approximation, Chi-Squared test, and Fisher’s Exact test to determine the statistical significance of all data points. Statistical significance was set at p<0.05.

## Results

OSP demographics include an average age of 49.4 years old, 49.3% female, 50.7% male, 61.8% current or have a history of smoking, 26.1% diagnosed with DM, 63.8% diagnosed with HTN, 23.2% treated with DM medication, and 58% treated with an antihypertensive (Table 2). On average, an OSP visited the clinic four times (Table 3). Demographics of FSP include average age of 60.1 years old, 33.8% female and 66.2% male, 52.9% are current or have a history of smoking, 36.8% were diagnosed with DM, 97.1% with HTN, 35.3% with DM medication, and 97.1% were treated with an antihypertensive (Table 2). On average, an FSP visited the clinic 31.2 times, and an OSP visited an average of 4 times (Table 3). There was a statistically significant difference between the number of OSP and FSP with HTN (p<0.0001) and treated with anti-hypertensives (p<0.0001). There was no statistically significant difference between FSP and OSP with DM (p=0.1781) or taking medication for DM (p=0.1192).

**Table 2:** Summary of Cass Clinic patients *2-9 visits; **>9 visits; p<0.05 is statistically significant OSP: occasionally seen patient, FSP: frequently seen patient, DM: diabetes mellitus, HTN: hypertension

Variable	All patients N(N%)	OSP* N(N%)	FSP** N(N%)	p-value
Number of patients	137	69	68	
Age	54.7 + 12.56	49.4 + 13.66	60.1 + 8.51	<0.0001
Sex				0.0666
Female	57 (41.6)	34 (49.3)	23 (33.8)	
Male	80 (58.4)	35 (50.7)	45 (66.2)	
Race				0.6795
African American	87 (87.9)	38 (86.4)	49 (89.1)	
White	12 (12.1)	6 (13.6)	6 (10.9)	
Ever smoker				0.2982
Yes	78 (57.4)	42 (61.8)	36 (52.9)	
No	58 (42.6)	26 (38.2)	32 (47.1)	
Diabetic				0.1781
Yes	43 (31.4)	18 (26.1)	25 (36.8)	
No	94 (68.6)	51 (73.9)	43 (63.2)	
Hypertension				<0.0001
Yes	110 (80.3)	44 (63.8)	66 (97.1)	
No	27 (19.7)	25 (36.2)	2 (2.9)	
Patient takes DM medication				0.1192
Yes	40 (29.2)	16 (23.2)	24 (25.3)	
No	97 (70.8)	53 (76.8)	44 (64.7)	
Patient takes HTN medication				<0.0001
Yes	106 (77.4)	40 (58)	66 (97.1)	
No	31 (21.6)	29 (42)	2 (2.9)	

**Table 3:** Statistical summary for all clinic patients by the use rate *2-9 visits; **>9 visits; p<0.05 is statistically significant SBP: systolic blood pressure, DBP: diastolic blood pressure, BMI: body mass index, A1c: hemoglobin A1c

	All patients	All patient’s mean + SD	OSP* N	OSP* mean + SD	FSP** N	FSP** mean +SD	p-value
Initial visit SBP (mmHg)	130	147.3 + 19.48	64	141.5 + 19.76	66	152.8 + 17.64	0.0008
Initial visit DBP (mmHg)	129	90.9 + 13.11	64	87.2 + 12.55	65	94.5 + 12.71	0.0017
Initial visit BMI	84	31.7 + 7.56	49	30.68 + 7.48	35	33.14 + 7.54	0.1405
Initial A1c	32	9.3 + 2.71	10	9.12 + 3.41	22	9.38 + 2.41	0.4816
Most recent visit SBP (mmHg)	134	135.2 + 16.4	66	135.9 + 15.93	68	134.5 + 16.93	0.5983
Most recent visit DBP (mmHg)	134	82.3 + 10.71	66	84 + 11.04	68	80.7 + 10.2	0.0816
Most recent visit BMI	109	31.25 + 7.346	51	31.5 + 7.65	58	31.03 + 7.13	0.8701
Most recent A1c	25	7.65 + 1.90	5	6.84 + 0.063	20	7.86 + 2.07	0.5681
Visit count	137	17.5 + 18.27	69	4 + 2.32	68	31.2 + 17.11	<0.0001
SBP Delta	128	−12.1 + 20.05	62	−5.5 + 19.45	66	−18.3 + 18.71	0.0002
DBP Delta	127	−8.7 + 14.7	62	−3 + 13.57	65	−14.2 + 13.71	<0.0001
A1c Delta	25	−1.4 + 1.35	5	−0.5 + 1.11	20	−1.7 + 1.32	0.0794
BMI Delta	73	−0.13 + 2.084	42	−0.18 + 2.19	31	−0.06 + 1.967	0.4637

Among all patients, SBP decreased by an average of 12.1 mmHg (95% CI [−15.57, −8.63]) and DBP decreased by 8.7 mmHg (95% CI [−11.25, −6.15]) between the first and most recent visit. OSP had an average SBP decrease of 5.5 mmHg (95% CI [−10.34, −0.659]) and a DBP of 3 mmHg (95% CI [−6.38, 0.38]). FSP had an average SBP decrease of 18.3 mmHg (95% CI [−22.81, −13.79]) and a DBP decrease of 14.2 mmHg (95% CI [−17.53, −10.87]). There was a statistically significant difference in the decrease in SBP and DBP between OSP and FSP (p<0.0001 and p=0.0002, respectively) (Table 3). There was no statistically significant difference in decreased HbA1c (p=0.0794). Patients with at least two recorded BMIs (n=73) saw a decrease of 0.13 kg/m^2^ (95% CI [−0.608, 0.348]). OSP had a decrease of 0.18kg/m^2^ (n=42) (95% CI [−0.842, 0.482]) and FSP had a decrease of 0.06kg/m^2^ (n=31) (95% CI [−0.752, 0.632]). The average BMI change stratified by OSP and FSP was not statistically significant (p=0.4637) (Table 3).

Patients were separated by a diagnosis of either HTN or DM and then further stratified by utilization of the clinic. The median visit count was 15 for patients diagnosed with HTN; the median visit count was 13 for patients diagnosed with DM; and the median visit count was 17.5 for patients diagnosed with DM and HTN. Disease-specific median visit counts were used to further analyze disease modification for patients with HTN or DM by once again classifying patients as OSP or FSP.

Overall, patients diagnosed with HTN (n=110) had SBP decreased by 14.1 mmHg (95% CI [−17.95, −10.26]) and DBP decreased by 9.8 mmHg (95% CI [−12.67, −6.94]). OSP (two to 15 visits) with HTN had an average SBP decrease of 10.2 mmHg (95% CI [−14.24, −6.16]) and DBP of 5.3 mmHg (95% CI [−8.13, −2.47]), while FSP (>15 visits) with HTN had an average SBP decrease of 18.2 mmHg (95% CI [−21.70, −14.70]) and DBP of 14.8 mmHg (95% CI [−17.42, −12.18]). The difference in the decrease in SBP and DBP in OSP and FSP with HTN was statistically significant (p=0.0426 and p=0.0010, respectively) (Table 4). Furthermore, the average age of FSPs diagnosed with HTN was 60.4 years old; for OSPs, the average age was 55.2 years old (p=0.0025) (Table 4).

**Table 4:** Statistical summary for Cass Clinic patients with diagnosis of hypertension *2-15 visits; **>15 visits; p<0.05 is statistically significant SBP: systolic blood pressure, DBP: diastolic blood pressure, BMI: body mass index, A1c: hemoglobin A1c

	All patients N	All patients mean + SD	OSP* N	OSP* mean + SD	FSP** N	FSP** mean +SD	p-value
Initial visit SBP (mmHg)	107	151.5 + 17.97	56	149.5 + 19.01	51	153.7 + 16.68	0.2368
Initial visit DBP (mmHg)	106	93.2 + 12.67	56	91.1 + 11.91	50	95.6+ 13.19	0.0990
Initial visit BMI	68	32.94 + 7.34	41	33.29 + 7.40	27	32.4 + 7.35	0.7174
Initial A1c	28	9.1 + 2.54	11	8.41 + 2.93	17	9.55 + 2.23	0.1264
Most recent visit SBP (mmHg)	109	137.6 + 16.37	56	139.7 + 17.97	53	135.5 + 14.34	0.0633
Most recent visit DBP (mmHg)	109	83.5 + 10.73	56	85.6 + 11.86	53	81.2 + 8.98	0.0256
Most recent visit BMI	88	32.17 + 7.15	44	33.24 + 7.03	44	31.11 + 7.19	0.2248
Most recent A1c	22	7.54 + 1.76	7	6.56 + 0.45	15	7.99 + 1.96	0.1116
Visit count	110	20.2 + 18.51	57	6 + 4.14	53	35.4 + 15.58	<0.0001
SBP Delta	106	−14.1 + 20.2	55	−10.2 + 21.21	51	-18.2 + 18.38	0.0426
DBP Delta	105	−9.8 + 14.98	55	−5.3 + 14.78	50	−14.8 + 13.69	0.0010
A1c Delta	22	−1.5 + 1.33	7	−0.9 + 0.98	15	−1.8 + 1.39	0.1299
BMI Delta	59	−0.08 + 2.21	35	−0.13 + 2.33	24	0.01 + 2.07	0.2336

There were 43 patients with documented DM. Only 25 had at least two HbA1c recordings for analysis. Among those 25 patients, there was a decrease of 1.4% in HbA1c. OSP (two to 13 visits) with DM had an HbA1c decrease of 0.50% (n=5) and FSP (>13 visits) with DM had a decrease of 1.7% (n=20). The average HbA1c decrease between OSP and FSP was not statistically significant (p=0.1733) (Table 5). However, the SBP decrease among DM patients was significant. Among all patients with DM, SBP decreased by 12.7 mmHg (95% CI [−19.21, −6.19]) and DBP decreased by 6.4 mmHg (95% CI [−11.12, −1.68]). OSP with DM had an average SBP decrease of 5.2 mmHg (95% CI [−14.54, 4.14]) and DBP of 4 mmHg (95% CI [−11.77, 3.77]), while FSP with DM had an average SBP decrease of 20.9 mmHg (95% CI [−28.65, −13.15]) and DBP of 9.1 mmHg (95% CI [−14.11, −4.09]). The difference in the decrease in SBP between OSP and FSP was statistically significant (p=0.0162), but was not for DBP (p=0.2867) (Table 5). The difference in BMI between OSP with DM, which decreased 0.73 kg/m^2^, and FSP with DM, which decreased by 0.67 kg/m^2^ was not statistically significant (p=0.4285) (Table 5).

**Table 5:** Statistical summary for Cass Clinic patients with diagnosis of type II diabetes mellitus *2-15 visits; **>15 visits; p<0.05 is statistically significant SBP: systolic blood pressure, DBP: diastolic blood pressure, BMI: body mass index, A1c: hemoglobin A1c

	All patients N	All patients mean + SD	OSP* N	OSP* mean + SD	FSP** N	FSP** Mean +SD	p-value
Initial visit SBP (mmHg)	42	147.8 + 20.44	22	143.3 + 20.88	20	152.7 + 19.29	0.1415
Initial visit DBP (mmHg)	42	88.2 + 11.09	22	87.3 + 13.72	20	89.3 + 7.43	0.5607
Initial visit BMI	29	34.42 + 8.29	18	35.38 + 8.89	11	32.85 + 7.32	0.4364
Initial A1c	30	8.99 + 2.71	12	8.42 + 2.78	18	9.37 + 2.29	0.1604
Most recent visit SBP (mmHg)	43	135 + 15.72	22	138.1 + 14.64	21	131.7 + 16.46	0.1803
Most recent visit DBP (mmHg)	43	81.8 + 9.97	22	83.3 + 11.23	21	80.2 + 8.46	0.3244
Most recent visit BMI	35	32.97 + 8.03	17	16.9 + 2.61	18	31.18 + 7.81	0.1785
Most recent A1c	24	7.47 + 1.71	8	6.68 + 0.534	16	7.87 + 1.96	0.2434
Visit count	43	19.5 + 17.25	22	5.2 + 3.62	21	34.5 + 12.99	<0.0001>
SBP Delta	42	−12.7 + 21.53	22	−5.2 + 22.34	20	−20.9 +17.68	0.0162
DBP Delta	42	−6.4 + 15.62	22	−4 + 18.59	20	−9.1 + 11.43	0.2867
A1c Delta	24	−1.4 + 1.37	8	−0.9 + 1.22	16	−1.7 + 1.4	0.1733
BMI Delta	26	−0.14 + 2.278	15	−0.73 + 1.995	11	−0.67 + 2.48	0.4285

## Discussion

Cass Clinic primarily treats patients with chronic diseases, including HTN, DM, and obesity. A majority of patients in our study had been diagnosed with HTN, DM, or both. Our study demonstrates that patients overall saw a decrease in their systolic and diastolic blood pressures. Among all patients, SBP decreased by an average of 12.1 mmHg and DBP decreased by 8.7 mmHg between the first and most recent visit. This finding is clinically relevant as studies have shown a 5 mmHg reduction in systolic blood pressure reduces the risk of major cardiovascular events by about 10%, irrespective of previous diagnoses of cardiovascular disease and even at normal or high-normal blood pressure values [10]. Patients who were seen more frequently had a greater reduction in both SBP and DBP, compared with those less frequently seen in the clinic. This finding was confirmed when patients with a formal diagnosis of HTN were analyzed separately. The more significant decrease in blood pressure in FSP may be attributed to a number of factors. First, the average age of FSP was higher than OSP by 10.7 years overall, and by 5.2 years in those diagnosed with hypertension. Older age is often associated with more advanced stages of chronic disease and thus may leave more room for improvement. For example, the average SBP and DBP measured at the FSPs’ initial visits were higher than the OSPs’ in our patients overall; however, this difference did not hold true for patients with HTN. It is also possible that those who frequent the clinic more often may inherently be more motivated to improve their health outcomes and be more adherent to their medications and lifestyle adjustments.

Patients with DM had an overall decrease in their initial versus final HbA1c values regardless of the visit frequency. While there was no statistically significant difference in the decrease in average HbA1c between FSP and OSP, a clinically significant improvement was present in both groups. OSP had an average decrease in HbA1c by 0.50% and FSP by 1.7%. Based on the ADA and National Institute for Health and Clinical Excellence treatment guidelines, 0.5% HbA1c is considered a clinically significant change [11,12]. It has been reported that an HbA1c decrease of 1% is associated with an 18% decrease in cardiovascular risk and a 37% reduction in microvascular complications [13,14]. The lack of statistical significance may be due to the smaller sample size (n=25) of patients who had two recorded HbA1c values in their records. Cass Clinic’s EMR does not contain a designated section for HbA1c to be charted (unlike BP, HR, etc.), and volunteers are often asked to enter the data into the patient’s note. It is likely that not all values were recorded, given the relatively higher number of patients diagnosed with DM (n=43).

It is also worth noting that in those diagnosed with DM, FSP saw a statistically significant decrease in SBP compared to OSP. However, OSP still had a clinically significant decrease of 5.2 mmHg in SBP. Diabetic patients with uncontrolled HTN have a substantially higher risk of developing coronary artery disease, retinopathy, stroke, and renal disease, compared with those only diagnosed with diabetes [15]. Lowering BP in diabetic patients resulted in a decreased risk of cardiovascular events, coronary heart disease, stroke, albuminuria, and retinopathy, ultimately improving the mortality of these patients [16,17].

The Cass clinic primarily manages obesity through counseling and its partnership with FreshRx and local food banks. While there was no significant difference in BMI between FSP and OSP, all patients did have an average decrease of 0.13 kg/m^2^ in their BMI. Notably, patients with DM had the greatest decrease in BMI. This may be attributed to the rigorous nutrition counseling and diabetes management education provided by the volunteer diabetic nurse practitioner in the clinic and the medical students. Proper nutrition therapy is essential to the management of DM and HTN. Cass Clinic’s partnership with FreshRx further reiterates this point by providing patients with the opportunity to obtain fresh produce and participate in cooking classes. The lack of statistical significance in regards to BMI may be twofold-for some patients, the goal may not be weight loss but weight maintenance and improving nutrition overall by choosing healthier foods. Additionally, BMI is based on weight, which can be impacted by the clothes a patient is wearing. It is possible that weight was not accurately measured and recorded, which can impact the calculation of BMI. Moving forward, the clinic would benefit from a standardized way to measure weight and more close follow-up of patients who are obese. Furthermore, partnering with other community organizations to promote exercise and healthy eating may also help improve the clinic’s obesity management.

Controlling BP in hypertensive patients (maintaining <130/80 mmHg) continues to challenge the U.S. healthcare system for decades despite effective treatments and national campaigns prioritizing BP control in those with HTN and DM [18]. The biennial National Health and Nutrition Examination Survey (NHANES) demonstrated no significant change in mean SBP or DBP among hypertensive patients receiving pharmacologic intervention between 1999 and 2016 [19]. Overall, NHANES shows that over a four-year period from 2013 to 2016, the average age-adjusted hypertensive patient saw average SBP and DBP increase [20]. These findings contrast with those of Cass Clinic’s over a similar time period, where hypertensive patients had an initial recorded average BP of 151.5/93.2 mmHg, with a 12.1 mmHg reduction in SBP and an 8.7 mmHg reduction in DBP. Additionally, the average hypertensive FSP, where the greatest improvement in BP was seen, was 60.1 years old and African American. These demographics nationally are challenging to treat (i.e., African American, age ≥ 60), since they have shown no consistent improvement in BP control based on NHANES data from 1999 to 2016 [19]. Additional relevant demographic data including homelessness, poverty, no primary care provider, and uninsured are not actively tracked by Cass Clinic, but are demographics our clinic is designed to treat. These serve as additional barriers that often limit adherence and BP management and are hurdles our patients manage to overcome.

With a limited data set, we believe the clinic has demonstrated a meaningful improvement in BP management among patients with various social barriers to health, such as lack of insurance and transportation. We believe the clinic’s success in lowering both SBP and DBP and the statistical association with the number of clinic visits is largely due to the clinic’s holistic approach, which promotes treatment compliance by providing close follow-up and a multitude of services. Patients receive not only care for their chronic diseases but can have eye exams, referrals to psychiatric and dental services, free clothing and toiletries, and care for any other acute issues during the visit. The services and medications provided by Cass Clinic may have a direct relationship with decreased BP values in patients who were more frequently seen. Frequently seen patients may have their BP monitored more regularly by the clinic, allowing for more frequent medication adjustments and better control of the disease. In those not formally diagnosed with HTN but found to have elevated BPs in the clinic, effective counseling may have affected the BP outcomes seen in our study.

National data from NHANES show that even with these medications being widely available and effective, national BP target goals continue to fall short. This has been largely attributed to poor medication adherence [19]. Improved adherence in the ambulatory setting has most consistently been associated with simplified dosing regimens, while other interventions such as motivational strategies and complex interventions including home visits, work-site care, and telecommunication reminders have shown varied success [21]. Though it is unclear why Cass Clinic is demonstrating success in lowering BP in both OSP and FSP hypertensive patients, it is likely a combination of patient education, fewer available medication options, possibly translating to simpler regimens, and ease of access with a clinic model that is free for the patient and does not require insurance or appointments. Improved access may be contributing to increased patient clinic visits, which has been shown in national data to translate to a higher likelihood of BP control in hypertensive patients [20]. Future studies should be pursued to better elucidate the cause of these meaningful outcomes at the Cass Clinic.

This research was limited by the short study time period and the fact that we considered only two values for each surrogate marker in assessing the management of chronic diseases. Additionally, missing HbA1c recordings for DM patients limited our ability to make stronger conclusions about our data. At Cass Clinic, a standardized pre-volunteering meeting to train students on the EMR would likely help to alleviate inconsistent recording of data. Given our lack of statistically significant findings on BMI changes between the FSP and OSPs, additional training on effective counseling techniques regarding diet, exercise, and other lifestyle changes would not only be beneficial in lowering BMI but also BP and HbA1c. A controlled study comparing different counseling methods may be conducted to look for improvement in our surrogate markers. Finally, we believe that administering satisfaction surveys to all patients would help to improve the clinic experience, which may in turn be associated with better health outcomes for patients.

These encouraging findings support the notion that a student-run free clinic like Cass Clinic provides meaningful value for patients locally and serves as a community resource for managing BP, DM, and obesity. Literature exists on the importance of creating continuity of care for managing chronic disease states like HTN, especially the need for improved collaboration on the transition of care from emergency departments (ED) to primary care providers [22].

## Conclusions

Student-run free clinics like Cass Clinic may play a meaningful role, especially since Cass Clinic’s model is targeted toward patients who are uninsured, without a primary care physician, and may not be able to afford their medication. The population most likely to have undiagnosed or uncontrolled HTN and be reliant on ED resources for chronic disease management may be the same population targeted by a student-run free clinic. Moving forward, a retrospective cohort study of Cass Clinic patients and their utilization of ED resources for chronic disease management may provide further insight into this theory. Additionally, the proximity of Cass Clinic to a major hospital system, its accommodation of walk-ins, and its referral system to higher levels of care may further support the clinic’s role in this continuity of care.
